# Variation in the Surgical Care of Early Stage Melanoma Based on Surgical Subspecialty: Evaluation of Large Healthcare System

**DOI:** 10.1097/AS9.0000000000000650

**Published:** 2026-02-09

**Authors:** Kristel Lourdault, Arthur W. Cowman, Emiliano Reyes, Danny Lascano, Udai S. Sibia, Brett Crosby, Tyler Aguilar, Deanna Rider, Stacey Stern, Richard Essner

**Affiliations:** From the * Saint John’s Cancer Institute, Providence Health & Services, Division of Surgical Oncology, Borstein Family Melanoma Program, Laboratory of Cutaneous Oncology, Santa Monica, CA; † Providence Research Network, Providence Health System, Missoula, MT.

**Keywords:** melanoma, National Comprehensive Cancer Network, sentinel lymph node biopsy, surgical margin size, wide excision

## Abstract

**Objective:**

To investigate how different surgeons’ subspecialties comply with treatment guidelines for early stage melanoma.

**Background:**

The National Comprehensive Cancer Network developed evidence-based guidelines for surgical margins, depth of excision, and performance of sentinel lymph node biopsy (SLNB) for cutaneous melanoma care. It is unknown how surgeons of varying subspecialties follow these guidelines.

**Methods:**

We included patients with primary cutaneous melanoma who underwent wide excision (WE) in 2022 across our healthcare system. We collected patients’ clinicopathological characteristics, margin size, and depth of the WE, those who underwent SLNB and the treating surgeon’s subspecialty.

**Results:**

Among 838 patients, 58.8% had T1 melanoma, 22.2% T2, and 18.9% T3–T4. Surgical oncologists (SO) performed most procedures (56.4%) followed by general surgeons (GS, 20.1%), dermatologists (Derm, 13.5%), otolaryngologists (ENT, 5.4%), and plastic surgeons (PS, 4.7%). Compliance with the recommended surgical margin size was highest for T1 (81.7%) and T2 (92.6%) compared to T3–T4 stages (64.2%; *P* < 0.0001). By subspecialty, Derm had the highest compliance rate with the margin size recommendations (93.3%) followed by SO (86.9%), PS (70%), GS (64%), and ENT (56.5%; *P* < 0.0001). Excision depth compliance increased with increasing T stage (*P* < 0.0001): 79.9%, 91.6%, and 92% for T1, T2, and T3–T4, respectively. 93% of eligible patients (T1b–T4) underwent SLNB.

**Conclusions:**

Overall compliance with surgical margin size and excision depth was 80.8% and 84.8%, respectively. In our study, 93% of eligible patients underwent SLNB. Despite overall high compliance rates, we noted large differences in guideline compliance among the surgeons’ subspecialty.

## INTRODUCTION

Cutaneous melanoma is an increasing health concern in the United States, with 105,000 new cases and 8400 deaths expected in 2025.^1^ The standard of care for invasive cutaneous melanoma is wide excision (WE) of the primary tumor, frequently associated with sentinel lymph node biopsy (SLNB). WE includes the original biopsy site and surrounding normal tissue. Surgical margins should be radial and are measured by the surgeon using a ruler from the edge of the biopsy site.^2,3^ The surgical margin size is based on the primary tumor pathological characteristics as described in the initial biopsy pathology report, and also determines the predicted risk of sentinel lymph node metastases and guidelines for use of SLNB.^4^

The National Comprehensive Cancer Network (NCCN) guidelines for the surgical margin for primary melanoma WE are based on several landmark randomized clinical trials investigating optimal margin size and patient outcome.^2,5–8^ These guidelines have changed over time, from the historically 3–5 cm margins^9^ to the current 1–2 cm margins depending on the primary tumor thickness.^10^ Melanoma less than 1 mm thick (=T1) should be excised with a 1 cm surgical margin; those >1 and up to 2 mm thick (=T2) should be excised with surgical margins between 1 and 2 cm; and those >2 mm thick (T3 and T4) should be removed with 2 cm surgical margins. Cosmetic concerns, anatomic, or functional limitations, may require narrower margins, especially for tumors located on the hands, feet, or head and neck (H&N) area. Additionally, all invasive melanoma excisions should include the skin and all underlying subcutaneous tissues down to the fascia without removing the fascia.

SLNB is generally performed for lymph node staging, which determines the role of postadjuvant therapy. SLNB is recommended for patients whose risk of having a tumor-positive sentinel lymph node is estimated to be at least 5%–10%, which includes some patients with T1a and all those with T1b and thicker melanoma. High-risk features such as increased mitotic rate, lympho-vascular invasion or ulceration increase the risk of lymph node disease.^11^

Beginning in January 2023, the Commission on Cancer (CoC), which bases its guidelines on the NCCN recommendations, mandated surgeons to record 4: elements in the operative note (1) curative surgical intent, (2) primary tumor thickness (= Breslow thickness), (3) surgical margin size, and (4) excision depth (down to fascia). The operative note should document the reason for any deviation for guidelines.^6,10^ The goal of these CoC guidelines is to standardize patient care to improve outcomes.

The goal of our study was to evaluate compliance with the NCCN surgical guidelines for primary invasive melanomas in 2022, before the implementation of the new CoC documentation guidelines in 2023. To answer this question, we assessed how surgeons of different subspecialties, in our healthcare system, complied with surgical margin size, excision depth, and performance of SLNB.

## MATERIAL AND METHODS

### Study Design

We queried the electronic health record for patients with newly diagnosed cutaneous melanoma (see ICD-10 codes in Supplemental Table 1, https://links.lww.com/AOSO/A570) located in our integrated healthcare system from January 1 to December 31, 2022, who underwent WE ± SLNB (see CPT codes in Supplemental Table 1, https://links.lww.com/AOSO/A570).

The following variables were collected from the medical record: age at diagnosis, primary tumor site, Breslow thickness, ulceration status, and T stage of biopsy specimen, surgical margin size reported by the operating surgeon, excision depth, performance of SLNB, pathological margin status, cases that had second excisions, hospital location, and surgeons’ designated subspecialty.

Cases were excluded from analysis if the primary diagnosis was melanoma in situ or if they had one of the exclusion criteria described in Supplemental Figure 1, https://links.lww.com/AOSO/A572.

T stages were defined according to the eighth edition of the American Joint Committee on Cancer melanoma staging system.^12^ The NCCN guidelines are: 1 cm surgical margins for T1 melanoma, between 1 and 2 cm for T2 melanoma, and 2 cm for T3–T4 melanoma. NCCN recommends excision should be done down to the fascia without removing the fascia. Cases were considered complaint if the surgeon used the recommended surgical margin size, performed the WE down to the fascia and kept the fascia intact.

Surgeons’ subspecialty was categorized as surgical oncologists (SO), dermatologists (Derm), plastic surgeons (PS), otolaryngologists (ENT), and general surgeons (GS). Due to small numbers, vascular, and orthopedic surgeons were included in the GS group.

This study was approved by our system-based Institutional Review Board.

### Statistical Analysis

Baseline demographic, tumor, and surgical characteristics were summarized using descriptive statistics and compared between groups using either a χ^2^ or Fischer exact test for categorical variables, and a Kruskal–Wallis test for continuous variables. All analyses were performed using SAS software, version 9.3. All reported *P*-values were 2-sided, with a value of ≤0.05 considered significant.

## RESULTS

### Patient Characteristics

In our integrated healthcare system, we identified 838 patients with 855 melanomas; some patients had more than 1 primary melanoma removed during 2022. Each melanoma was considered a separate event for this study. Of the 855 cases, 58.8% were T1 (71% of T1a and 29% of T1b), 22.2% T2 and 18.9% T3–T4 melanoma (Table 1 and Supplemental Table 2, https://links.lww.com/AOSO/A571).

**TABLE 1.** Patient’s Characteristics

2022									
	T1		T2		T3, T4		Total		
Characteristic	n	%	n	%	n	%	n	%	*P*
Primary tumors	503	58.8%	190	22.2%	162	18.9%	855	100.0%	
Patients (unique by T stage)	492	58.7%	188	22.4%	158	18.9%	838	100.0%	
Age, years: mean (SD)	64.1 (13.9)		64.7 (14.7)		69.0 (14.1)		65.6 (14.2)		0.017*
Age group									0.0087†
18–59	156	31.0%	61	32.1%	34	21.0%	251	29.4%	
60–69	161	32.0%	52	27.4%	45	27.8%	258	30.2%	
70–79	123	24.5%	46	24.2%	44	27.2%	213	24.9%	
80+	63	12.5%	31	16.3%	39	24.1%	133	15.6%	
Breslow, mm: mean (SD)	0.58 (0.24)		1.46 (0.29)		4.72 (4.30)		1.55 (2.4)		<0.0001*
Ulceration									<0.0001†
Yes	14	2.8%	36	18.9%	87	53.7%	137	16.0%	
No	489	97.2%	154	81.1%	75	46.3%	718	84.0%	
Primary site									0.4687†
Head/Neck	103	20.5%	46	24.2%	41	25.3%	190	22.2%	
Trunk	211	41.9%	66	34.7%	59	36.4%	336	39.3%	
Extremity	189	37.6%	78	41.1%	62	38.3%	329	38.5%	
State									<0.0001‡
AK	3	0.6%	5	2.6%	5	3.1%	13	1.5%	
CA	296	58.8%	104	54.7%	88	54.3%	488	57.1%	
MT	7	1.4%	7	3.7%	5	3.1%	19	2.2%	
OR	142	28.2%	37	19.5%	26	16.0%	205	24.0%	
TX	5	1.0%	7	3.7%	8	4.9%	20	2.3%	
WA	50	9.9%	30	15.8%	30	18.5%	110	12.9%	
Specialty (procedure performed by)									<0.0001†
Dermatology (n = 29)	115	22.9%	0	0.0%	0	0.0%	115	13.5%	
General surgery (n = 52)	90	17.9%	48	25.3%	34	21.0%	172	20.1%	
Otolaryngology (n = 21)	18	3.6%	14	7.4%	14	8.6%	46	5.4%	
Plastic surgery (n = 15)	27	5.4%	6	3.2%	7	4.3%	40	4.7%	
Surgical oncology (n = 19)	253	50.3%	122	64.2%	107	66.0%	482	56.4%	
Surgical margins size									<0.0001†
<1 cm	23	4.6%	5	2.6%	1	0.6%	29	3.4%	
=1 cm	411	81.7%	62	32.6%	19	11.7%	492	57.5%	
1–2 cm	38	7.6%	53	27.9%	21	13.0%	112	13.1%	
=2 cm	13	2.6%	61	32.1%	104	64.2%	178	20.8%	
>2 cm	2	0.4%	0	0.0%	2	1.2%	4	0.5%	
Unknown	16	3.2%	9	4.7%	15	9.3%	40	4.7%	
Excision down to fascia									<0.0001†
Yes	402	79.9%	174	91.6%	149	92.0%	725	84.8%	
No	92	18.3%	11	5.8%	7	4.3%	110	12.9%	
Unknown	9	1.8%	5	2.6%	6	3.7%	20	2.3%	
Fascia removed									<0.0001†
Yes	21	4.2%	16	8.4%	28	17.3%	65	7.6%	
No	9	1.8%	6	3.2%	6	3.7%	21	2.5%	
Unknown	473	94.0%	168	88.4%	128	79.0%	769	89.9%	
SLNB performed									<0.0001†
Yes	231	45.9%	180	94.7%	152	93.8%	563	65.8%	
No	272	54.1%	10	5.3%	10	6.2%	292	34.2%	
Blue dye used for SLNB (n = 563)	n = 231		n = 180		n = 152		n = 563		<0.0001†
Yes	168	72.7%	100	55.6%	77	50.7%	345	61.3%	
No	63	27.3%	80	44.4%	75	49.3%	218	38.7%	
Probe used for SLNB (n = 563)	n = 231		n = 180		n = 152		n = 563		0.0014†
Yes	228	98.7%	171	95.0%	140	92.1%	539	95.7%	
No	3	1.3%	9	5.0%	12	7.9%	24	4.3%	
Residual melanoma									<0.0001†
Yes	98	19.5%	67	35.3%	77	47.5%	242	28.3%	
Yes, in situ	91	18.1%	28	14.7%	14	8.6%	133	15.6%	
No	314	62.4%	95	50.0%	71	43.8%	480	56.1%	
Positive margins									0.2477‡
Yes	9	1.8%	1	0.5%	6	3.7%	16	1.9%	
Yes, in situ	12	2.4%	3	1.6%	2	1.2%	17	2.0%	
No	482	95.8%	186	97.9%	154	95.1%	822	96.1%	
Re-excision after positive margins (n = 33)	n = 21		n = 4		n = 8		n = 33		0.8429‡
Yes	16	76.2%	3	75.0%	7	87.5%	26	78.8%	
No	5	23.8%	1	25.0%	1	12.5%	7	21.2%	
Use of CoC form									0.252†
Yes	41	8.2%	23	12.1%	17	10.5%	81	9.5%	
No	462	91.8%	167	87.9%	145	89.5%	774	90.5%	

* Kruskall–Wallis test.

† χ^2^ test.

‡ Fisher exact test.

The average age of patients is 65.6 ± 14.2 years old. Patients with T1 and T2 were younger compared to those with T3–T4 melanoma (*P* = 0.017). The average Breslow thickness of the cohort is 1.55 ± 2.4 mm. Sixteen percent of the patients had an ulcerated primary tumor; with an increasing rate of ulceration with increasing T stage: 2.8%, 18.9%, and 53.7% for patients with T1, T2, and T3–T4 melanoma, respectively (*P* < 0.0001). The primary tumors were located on the trunk, extremities, and H&N region in 39.3%, 38.5%, and 22.2% of cases, respectively (Table 1).

Our integrated healthcare system includes more than 50 hospitals across 6 states: Alaska, Washington, Oregon, California, Texas, and Montana. It includes a wide range of hospitals from small community clinics to large urban cancer centers. Most surgeries were performed in California (57.1%) followed by Oregon (24%), Washington (12.9%), Texas (2.3%), Montana (2.2%), and Alaska (1.5%) (Table 1).

Within our healthcare system, several surgical subspecialties treat melanoma patients, including SO, Derm, PS, ENT, and GS. Despite representing only 14% of the surgeons in our study, SO performed more than half of the cases (56.4%). GS who represents 38% of the surgeons in this study, did 20.1% of the cases. Derm only manages T1 melanoma. As expected, all cases treated by ENT were tumors located in the H&N region. PS mainly treated patients with T1a melanoma (65%) (Table 1).

### Surgical Excision Margin Width

**FIGURE 1.**
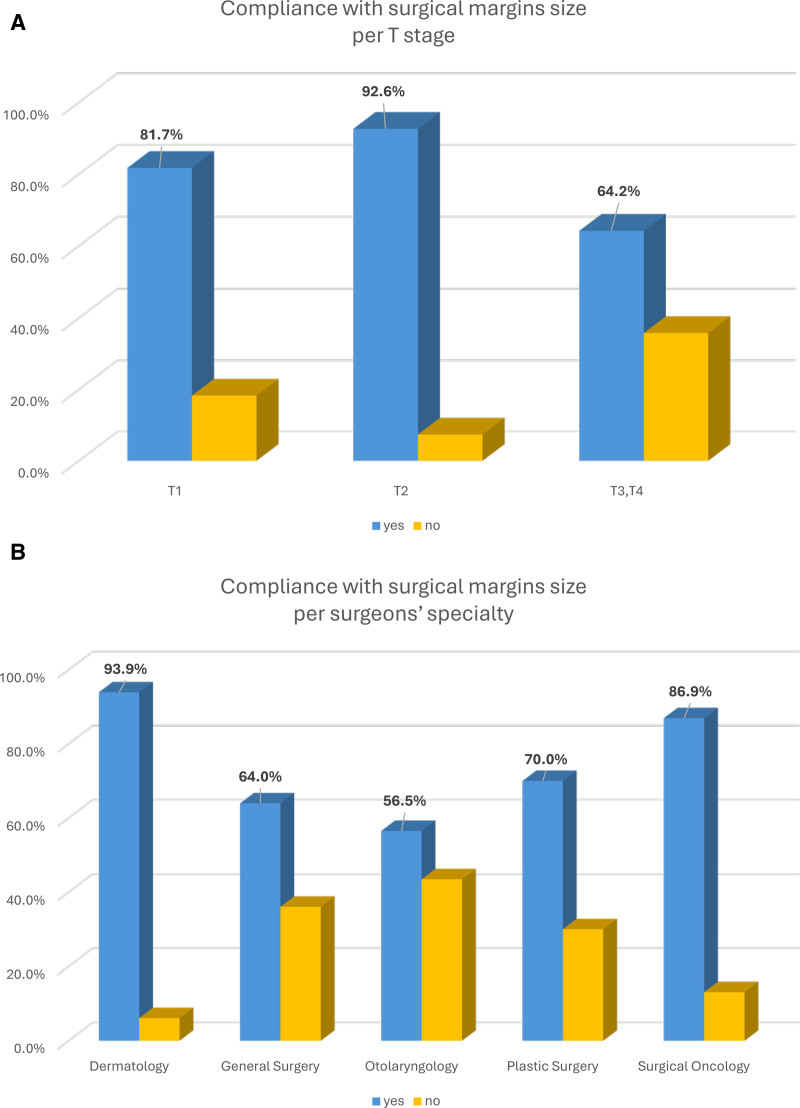
Compliance with the size of the surgical margins (A) per T stage and (B) per surgeons’ subspecialty. In blue are cases that complied with the size for the surgical margins and in yellow those that didn’t.

In our cohort, 81.7% of T1, 92.6% of T2, and 64.2% of T3–T4 melanomas were excised in compliance with NCCN surgical margin size guidelines (Fig. 1A). The overall compliance rate with surgical margin size was 80.8%. A total of 7.2% of cases had surgical margins narrower than the recommended guidelines. Conversely, 6.7% of cases (mainly T1) had surgical margins larger than the recommended guidelines.

By subspecialty, Derm had the highest compliance rate with recommended surgical margin size (93.3%) followed by SO (86.9%), PS (70%), GS (64%), and ENT (56.5%; *P* < 0.0001) (Fig. 1B). Overall, the compliance with the surgical margin size decreased as the T stage increased (Supplemental Figure 2, https://links.lww.com/AOSO/A573).

Out of 69 patients with surgical margins smaller than guidelines: 23 had T1, 5 T2, and 41 T3–T4 melanomas. Most (32) were located in the H&N region, then trunk (15), and extremities (22). SO performed most cases (40) followed by GS (13), ENT (6), PS (6), and Derm (4). Twenty patients had narrower surgical margins due to cosmetic or anatomical reasons described by the surgeons—13 of them were located in the H&N region—5 patients were enrolled in a surgical margin size clinical trial (MelMart), and 1 refused the recommended surgical margin size for cosmetic concerns. No explanation for the deviation was mentioned for the remaining cases.

Out of 55 patients with surgical margins wider than the guidelines: 53 were T1 and 2 were T3 cases. Almost half of them (25) were located on the trunk, 16 on an extremity, and 14 on the H&N region. The majority of these cases were performed by GS (24) and SO (18). No explanation was provided for why the surgeon deviated from the guidelines.

Of the entire cohort, 33 (3.8%) patients had tumor-positive surgical margins after WE surgery, with melanoma in situ present at the surgical margins in half of the cases (Table 1). Among them, 26 had a second excision, and 7 did not: 2 because they started systemic therapy and 5 were clinically followed (Table 1).

Among the 69 patients with narrow surgical margins, 7 (10.1%) had tumor-positive margins, and 6 of them had additional surgery. Among the 53 patients with wide margins, 1 required a skin graft, and 5 had minor wound complications such as bleeding or hematoma.

### Depth of Excision

**FIGURE 2.**
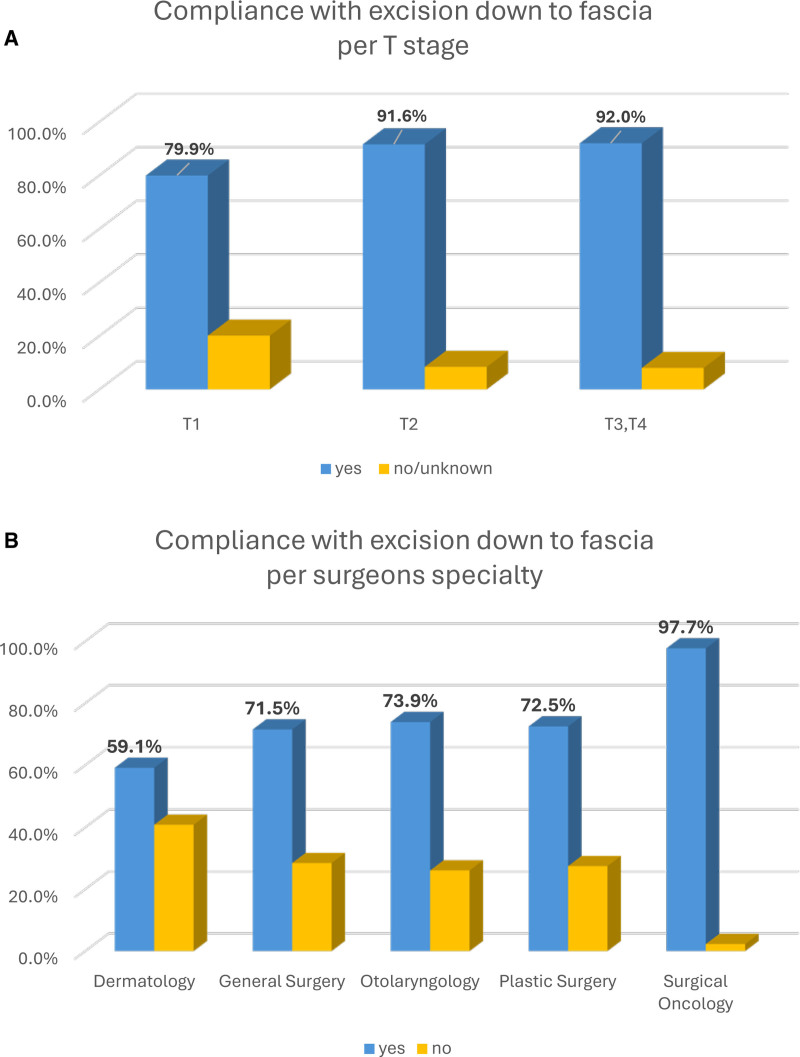
Compliance with the depth of excision (A) per T stage and (B) per surgeons’ subspecialty. In blue are cases that complied with the depth of excision down to fascia and in yellow those that didn’t.

Excision was performed down to fascia in 79.9%, 91.6%, and 92.0% of cases in T1, T2, and T3–T4, respectively (Table 1 and Fig. 2A). By subspecialty, SO had the highest compliance rate with excision depth guidelines (97.7%) followed by ENT (73.9%), PS (72.5%), GS (71.5%), and Derm (59.1%) (Fig. 2B).

The fascia was removed in 7.6% of the cases; in 4.2% of T1, 8.4% of T2, and 17.3% of T3–T4 (Supplemental Figure 3A, https://links.lww.com/AOSO/A574). By subspecialty, ENT had the highest non-compliance rate with keeping the fascia intact (19.6%) followed by PS (15%), GS (14%), and SO (5.4%) (Supplemental Figure 3B, https://links.lww.com/AOSO/A574).

Fascia was removed in 65 patients: in 10 patients due to concerns about tumor infiltrating the deep layers of the skin, down to the fascia and/or muscle; in 6 due to cosmetic reasons, such as maintaining the shape of an ear; and 4 had amputations of digits. For the remaining 45 patients, there was no explanation for why the fascia was removed.

### Sentinel Lymph Node Biopsy

**FIGURE 3.**
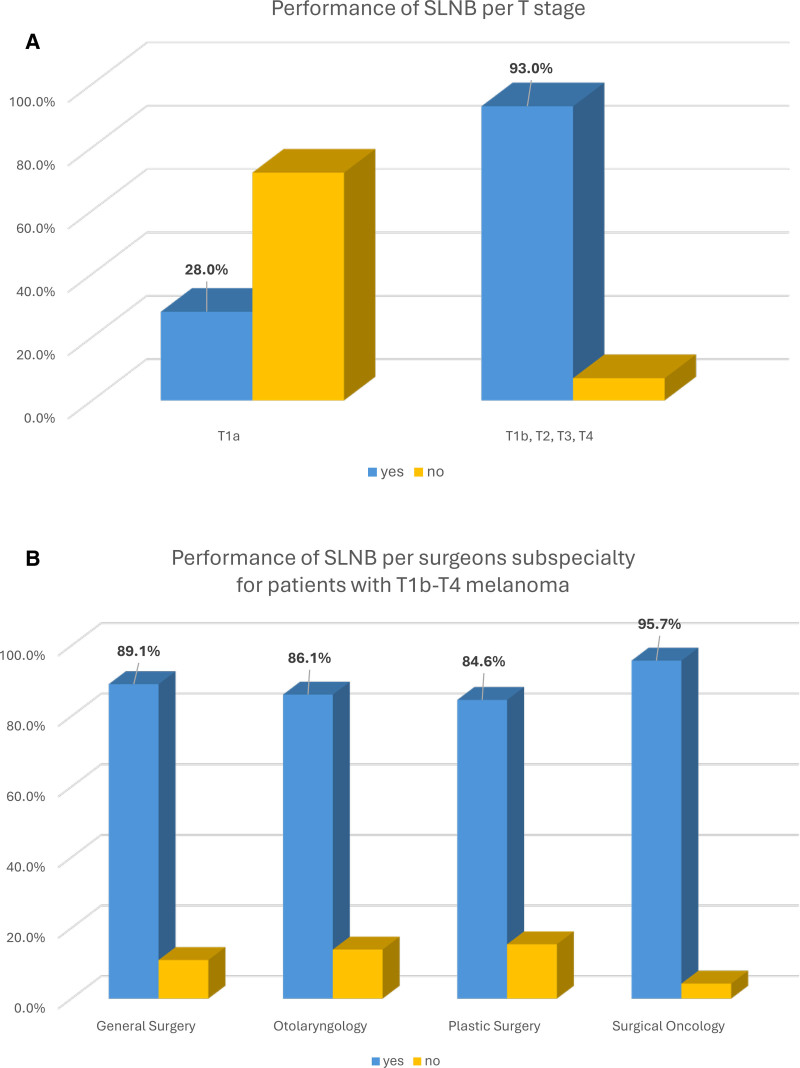
Performance of SLNB per patient’s T stage (A) per T stage and (B) per surgeons’ subspecialty. In blue are cases that underwent SLNB and in yellow those that didn’t.

SLNB was performed in 93.8% of T3–T4, 94.7% of T2, and 45.9% of T1 cases (28% in T1a and 89.7% in T1b) (Table 1 and Supplemental Table 2, https://links.lww.com/AOSO/A571). Overall, 93% of patients with T1b–T4 melanoma underwent SLNB (Fig. 3A).

SO performed SLNB in 95.7%, GS in 89.1%, ENT in 86.1%, and PS in 84.6% of patients with T1b and above melanoma (Fig. 3B). Out of the 115 cases treated by Derm, only one was eligible for SLNB (T1b), but didn’t undergo SLNB because it was characterized as low-risk by a prognostic test.

We considered cases with patients who underwent lymphoscintigraphy, but no lymph node was detected or collected as compliant with the guidelines. Hence, only 35 eligible patients didn’t undergo SLNB: 13 due to patient age and/or comorbidities, 7 refused SLNB and, 8 were not offered SLNB because categorized as low-risk. No reason why the patient didn’t undergo SLNB was listed for the remaining 7 patients.

Conversely, 100 patients with T1a underwent SLNB; 41 due to high-risk factors such as the presence of lympho-vascular invasion and/or increased mitotic rate. For the remaining 59 patients, no reason for SLNB was reported in the medical record.

## DISCUSSION

Surgical excision of the primary tumor is the initial approach to treat cutaneous melanoma. Several randomized clinical trials^2,5–8,10^ over the past 20 years have resulted in the current 1–2 cm margins guidelines^6^ depending on the thickness of the primary tumor. None of these trials showed a survival benefit from the historic wide margins. The international prospective randomized MelMart study is evaluating the utility of 1 versus 2 cm surgical margin size for all melanoma >1 mm in thickness (NCT02385214). The results of this trial may again alter recommendations for surgical margins.

Despite 80.8% compliance rate with surgical margin size guidelines, we noticed large differences among the T stage and the surgeons’ subspecialty. Compliance with the surgical margin size is highest with T1 (81.7%) and T2 (92.6%) compared to T3–T4 melanomas (64.2%). These differences could be explained by the fact that T3–T4 calls for 2 cm margins that are not always easily manageable, especially for lesions located on the H&N region or distal extremities. This may partially explain why ENT had the lowest compliance rate (56.5%), as they typically only manage melanomas on the H&N region. However, when we compared the surgical margin size compliance amongst surgeons for melanoma located on the H&N region—independently of the stage—we observed higher compliance rates for Derm (80%) and SO (75%) compared to GS (63.6%), ENT (52.2%), and PS (50%; *P* = 0.041) (Supplemental Figure 4A, https://links.lww.com/AOSO/A575). We noticed similar differences in surgical margin size compliance amongst surgeons’ subspecialties for melanoma located on the trunk and extremities (Supplemental Figures 4B, C, https://links.lww.com/AOSO/A575).

For all surgeons’ subspecialty the surgical margin size compliance is the lowest for T3–T4 cases (Supplemental Figure 2, https://links.lww.com/AOSO/A573). Surprisingly, ENT and GS have a high noncompliance rate for T1 melanomas because they tend to use wider surgical margins than recommended.

By subspecialty, Derm had the highest compliance rate with recommended surgical margin size (93.9%) followed by SO (86.9%), PS (70%), GS (64%), and ENT (56.5%; *P* < 0.0001) (Fig. 1B). The high rate of compliance for Derm might be associated with the fact that in this study they only treated patients with T1 melanoma that require only 1 cm surgical margins. The high rate of compliance for SO is most likely due to their specific training and the fact that they perform more melanoma surgeries per year. In our cohort, 19 SO performed in an average of 25 cases per surgeon compared to 3.3, 2.3, and 2.6 cases/year for GS, ENT, and PS, respectively. Higher volume of cases likely leads to better compliance with NCCN guidelines and lower volume by surgeons leads to lower compliance in other parts of the procedure (Fig. 2B).

Following surgical margins guidelines is a basic tenet of oncology care. Not following these guidelines leads to several issues; insufficient margins may result in a positive surgical margin, which have been associated with increased risk of local recurrence and potential distant disease.^13–15^ Additionally, when the initial removal is incomplete, patients require re-excision. Conversely, larger excision margins may require reconstruction with a skin graft or larger tissue flap increasing, in both cases, the cost and complexity of patient care.

In our study, 7.2% of patients had surgical margins smaller than the NCCN guidelines. Most of these cases were T3–T4, primarily located on the H&N region and were mainly performed by SO. The reason for deviation from the guidelines was often due to anatomical, cosmetic, or functional concerns. Among patients with narrow surgical margins, 10.1% had positive margins compared to 3.3% in the rest of the cohort (*P* = 0.013). Furthermore, 79% of patients with positive margins had re-excision surgery to clear the margins. Narrower margins were associated with increased risk of positive margins after WE surgery and need for a second excision, therefore likely increasing the risk of complications and the cost of patient care.^16^

In 84.8% of cases, surgeons complied with the excision depth, but in 7.6% of cases, the fascia was removed. Studies have shown that removing fascia doesn’t improve overall survival or decrease local recurrence and suggest that it may increase the risk of in-transit and nodal recurrence.^17–19^ Due to the short follow-up time, we could not assess survival and recurrence rates and couldn’t estimate the functional and cosmetic consequences of fascia removal during WE surgery.

The majority of eligible patients (93%) underwent SLNB. The recent study from Van Doren et al,^20^ showed 7.4% of patients with T1a underwent SLNB, significantly lower than in our study (28%), in which, most SLNB were performed by SO (90.6%). Our results are different from Van Doren et al, most likely because our health care system is made of community-based cancer centers where patients are referred specifically for SLNB, and patients often request the procedure despite the limited likelihood of a tumor-positive lymph node.^21,22^ It is important to notice that 41% of patients with T1a who underwent SLNB had high-risk factors such as increased mitotic rate and/or presence of LIV.

In 2020, the CoC created new standards for documenting primary cutaneous melanoma WE, with the goal of implementing these standards on January 1, 2023, and achieving 70% compliance that year. According to the CoC guidelines, each operative note should include the intent of the surgery, primary tumor thickness, the surgical margin size, excision depth (= down to fascia but not excised), and any deviation from the guidelines. Based on these criteria, in our healthcare system, 66.5% of cases complied with the CoC guidelines in 2022. After reviewing cases that didn’t comply with the guidelines, we identified 26 cases for which deviation is mentioned, and we considered these cases as compliant, bringing our rate of compliance to 69.6%.

A study from 2018, by Blakely et al,^23^ assessed the surgical margin size and SLNB compliance rate in their academic center. They reported high compliance rates for surgical margins ranging from 98.7% for thin melanoma to 92% for thick melanoma. These compliance rates could be due to the type of hospital patients were treated at (= academic referral center), and the limited number and training of the melanoma surgeons, surgical residents, and fellows. Moreover, excision depth wasn’t included in their study. If we only considered SO in our health system, the overall compliance rate is 84.8%. Our health system includes a variety of community and academic sites and may be more reflective of the true practice patterns in the United States and those physicians who treat melanoma, including Derm. A recent nationwide study from Katz et al,^24^ assessing compliance with the new CoC guidelines found a 53% compliance rate for melanoma WE—significantly lower than the 69.6% compliance observed in our study. Their more stringent definition of compliance: a case that meets both the technical and documentation requirements, could partially explain the difference in compliance rates between their study and ours.

This study is one of the first to assess an entire integrated health system compliance for guidelines of WE surgery in melanoma patients. In 2022, our compliance rates were 80.8% and 84.8% for the surgical margin size and depth of excision, respectively. Despite overall high compliance rates, our study demonstrated large differences among surgeons’ subspecialty in following guidelines, especially for the surgical margin size and excision depth. We suspect that the implementation in 2023 of the new CoC guidelines compliance for melanoma care will improve our numbers. Surgeons are required to document margins of excision, which we hope will translate to better surgical compliance with the guidelines, reducing the incidence of positive margins and need for additional surgery, and the burden of potentially increased morbidities and costs from a second procedure.

## ACKNOWLEDGMENTS

The authors would like to thank Kim Margolin, MD, for her careful and insightful review of the manuscript.

## Supplementary Material
