# Supplementary material for: Variation in the Surgical Care of Early Stage Melanoma Based on Surgical Subspecialty: Evaluation of Large Healthcare System
Source: Ann Surg Open. 2026 Feb 9;7(1):e650. doi: 10.1097/AS9.0000000000000650 (PMC13016181; doi:10.1097/AS9.0000000000000650)
Supplement: Supplementary file 1 [file as9-7-e650-s001.pdf]

Supplemental Table 1. ICD-10 and CPT codes used to define our study cohort.

ICD-10 codes		CPT codes			
**invasive melanoma**	**in situ melanoma**	**wide excision**			
C43	DO3	11600	11443	12045	150002
C43.0	D03.1	11601	11444	12046	150003
C43.1	D03.10	11602	11446	12047	150004
C43.10	D03.11	11603	11450	12051	15005
C43.11	D03.111	11604	11451	12052	15040
C43.111	D03.112	11606	11462	12053	15050
C43.112	D03.12	11620	11463	12054	15100
C43.12	D03.121	11621	11470	12055	15101
C43.121	D03.122	11622	11471	12056	15110
C43.122	D03.2	11623	12001	12057	15111
C43.2	D03.20	11624	12002	13100	15115
C43.20	D03.21	11626	12004	13101	15116
C43.21	D03.22	11640	12005	13102	15120
C43.22	D03.3	11641	12006	13120	15121
C43.3	D03.30	11642	12007	13121	15130
C43.30	D03.39	11643	12011	13122	15131
C43.31	D03.4	11644	12013	13131	15135
C43.39	D03.5	11646	12014	13132	15136
C43.4	D03.51	11400	12015	13133	15150
C43.5	D03.52	11401	12016	13151	15151
C43.51	D03.59	11402	12017	13152	15152
C43.52	D03.6	11403	12018	13153	15155
C43.59	D03.60	11404	12020	131600	15156
C43.6	D03.61	11406	12021	14000	15157
C43.60	D03.62	11420	12031	14001	15200
C43.61	D03.7	11421	12032	14020	15201
C43.62	D03.70	11422	12034	14040	15220
C43.7	D03.71	11423	12035	14041	15221
C43.70	D03.72	11424	12036	14060	15240
C43.71	D038	11426	12037	14061	15241
C43.72	D03.9	11440	12041	14301	15260
C43.8		11441	12042	14302	15261
C43.9		11442	12044	14350	15271

	SLNB
15272	38500
15273	38505
15274	38510
15275	38520
15276	38525
15277	38530
15278	38531
15570	38542
15572	38550
15574	38555
15576	38700
15600	38720
15610	38724
15620	38740
15630	38745
15650	38746
15730	38747
15731	38760
15733	38765
15734	38770
15736	38780
15738	
15740	
15750	
15756	
15757	
15758	
15760	
15770	
15775	
15776	
15777	
