# Supplementary material for: Variation in the Surgical Care of Early Stage Melanoma Based on Surgical Subspecialty: Evaluation of Large Healthcare System
Source: Ann Surg Open. 2026 Feb 9;7(1):e650. doi: 10.1097/AS9.0000000000000650 (PMC13016181; doi:10.1097/AS9.0000000000000650)
Supplement: Supplementary file 2 [file as9-7-e650-s002.pdf]

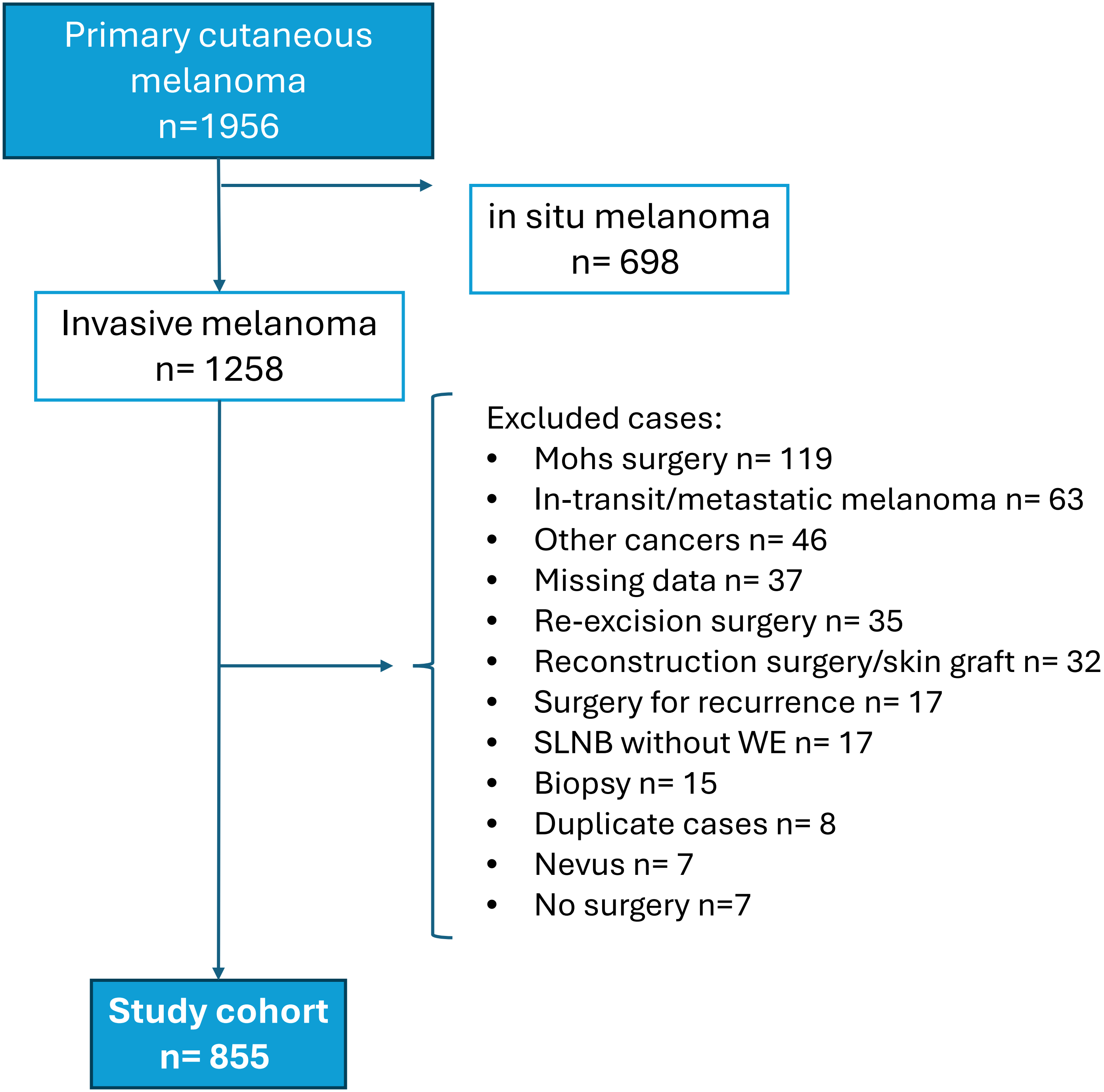
Primary cutaneous melanoma n=1956
in situ melanoma n= 698
Invasive melanoma n= 1258
Excluded cases:
• Mohs surgery n= 119
• In-transit/metastatic melanoma n= 63
• Other cancers n= 46
• Missing data n= 37
• Re-excision surgery n= 35
• Reconstruction surgery/skin graft n= 32
• Surgery for recurrence n= 17
• SLNB without WE n= 17
• Biopsy n= 15
• Duplicate cases n= 8
• Nevus n= 7
• No surgery n=7
Study cohort n= 855
