# Supplementary material for: Variation in the Surgical Care of Early Stage Melanoma Based on Surgical Subspecialty: Evaluation of Large Healthcare System
Source: Ann Surg Open. 2026 Feb 9;7(1):e650. doi: 10.1097/AS9.0000000000000650 (PMC13016181; doi:10.1097/AS9.0000000000000650)
Supplement: Supplementary file 3 [file as9-7-e650-s003.pdf]

Supplemental Table 2. Patients with T1a and T1b melanoma characteristics

Characteristic	T1a n	T1a %	T1b n	T1b %
**Primary tumors**	357	71.0%	146	29.0%
**Patients (unique by T Stage)**	346	70.2%	146	29.6%
**Age, years: mean (SD)**	64.4 (13.7)		64.4 (14.4)	
**Age group**				
18-59	107	30.0%	49	33.6%
60-69	126	35.3%	35	24.0%
70-79	82	23.0%	41	28.1%
80+	42	11.8%	21	14.4%
**Breslow, mm: mean (SD)**	0.46 (0.16)		0.87 (0.10)	
**Ulceration**				
Yes	0	0.0%	15	10.3%
No	357	100.0%	131	89.7%
**Primary Site**				
Head/Neck	71	19.9%	32	21.9%
Trunk	155	43.4%	56	38.4%
Upper Extremity	66	18.5%	34	23.3%
Lower Extremity	65	18.2%	24	16.4%
**State**				
AK	3	0.8%	0	0.0%
CA	214	59.9%	82	56.2%
MT	3	0.8%	4	2.7%
OR	119	33.3%	23	15.8%
TX	1	0.3%	4	2.7%
WA	17	4.8%	33	22.6%
**Specialty**				
Dermatology (n=29)	114	31.9%	1	0.7%
General Surgery (n=52)	53	14.8%	37	25.3%
Otolaryngology (n=21)	10	2.8%	8	5.5%
Plastic Surgery (n=15)	27	7.6%	0	0.0%
Surgical Oncology (n=19)	153	42.9%	100	68.5%
**Surgical Margins**				
<1cm	18	5.0%	5	3.4%
=1cm	299	83.8%	109	74.7%
1-2cm	22	6.2%	16	11.0%
=2cm	6	1.7%	9	6.2%
>2cm	12	3.4%	7	4.8%
unknown				
**Excision down to fascia**				
Yes	272	76.2%	130	89.0%
No	78	21.8%	14	9.6%
Unknown	7	2.0%	2	1.4%
**Fascia removed**				
Yes	11	3.1%	10	6.8%
No	339	95.0%	134	91.8%
Unknown	7	2.0%	2	1.4%
**SLNB performed (based on final N stage)**				
Yes	100	28.0%	131	89.7%

No	257	72.0%	15	10.3%
**Blue dye used for SLNB (n=564)**	n=	100	n=	131
Yes	76	76.0%	92	70.2%
No	24	24.0%	39	29.8%
**Probe used for SLNB (n=564)**	n=	100	n=	131
Yes	99	99.0%	129	98.5%
No	1	1.0%	2	1.5%
**Residual Melanoma**				
Yes	66	18.5%	32	21.9%
Yes, IS	60	16.8%	31	21.2%
No	231	64.7%	83	56.8%
**Positive Margins**				
Yes	7	2.0%	2	1.4%
Yes, IS	8	2.2%	4	2.7%
No	342	95.8%	140	95.9%
**Re-Excision after Positive Margins (n=21)**	*n= 15*		*n= 6*	
Yes	10	66.7%	6	100.0%
No	5	33.3%	0	0.0%
**Use of CoC form**				
Yes	27	7.6%	14	9.6%
No	330	92.4%	132	90.4%
