# Supplementary figures and images for: Variation in the Surgical Care of Early Stage Melanoma Based on Surgical Subspecialty: Evaluation of Large Healthcare System
Source: Ann Surg Open. 2026 Feb 9;7(1):e650. doi: 10.1097/AS9.0000000000000650 (PMC13016181; doi:10.1097/AS9.0000000000000650)

## Compliance with the surgical margin size per T stage and surgeons subspecialty

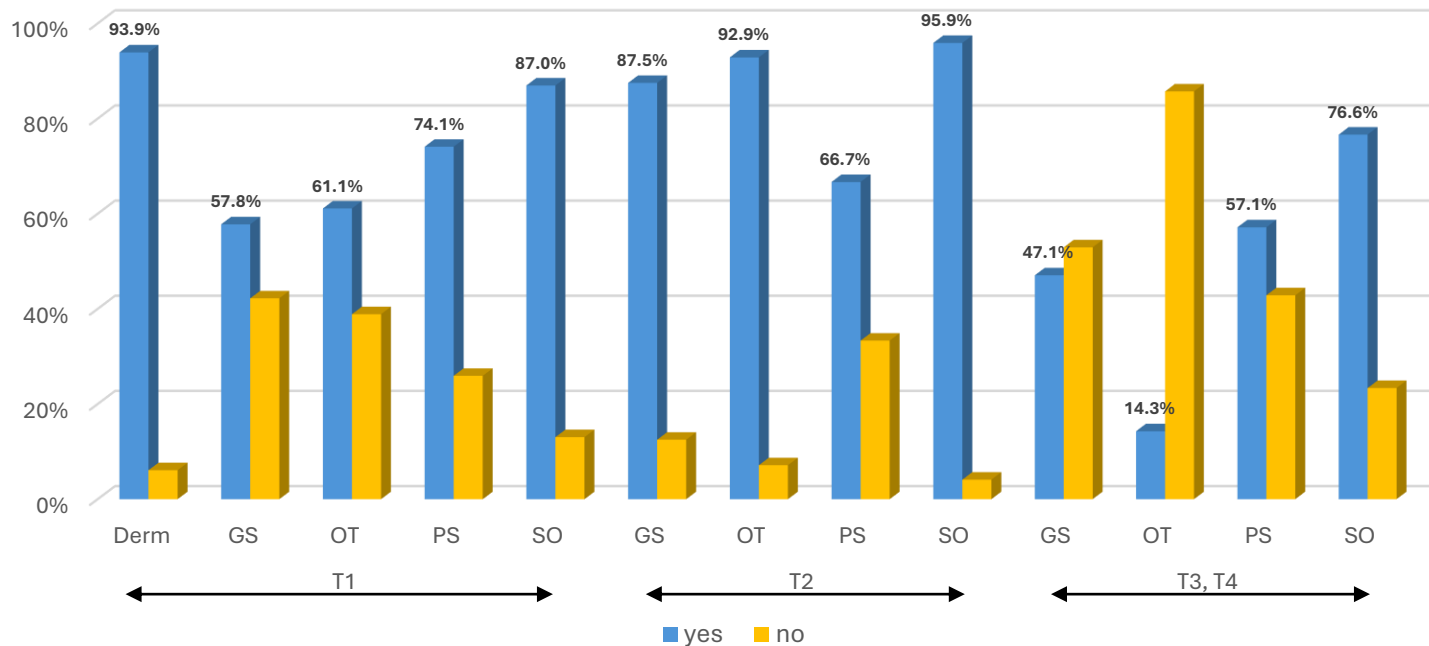

Supplement: Supplementary file 4 [file as9-7-e650-s004.pdf]
