# Supplementary material for: Variation in the Surgical Care of Early Stage Melanoma Based on Surgical Subspecialty: Evaluation of Large Healthcare System
Source: Ann Surg Open. 2026 Feb 9;7(1):e650. doi: 10.1097/AS9.0000000000000650 (PMC13016181; doi:10.1097/AS9.0000000000000650)
Supplement: Supplementary file 5 [file as9-7-e650-s005.pdf]

A

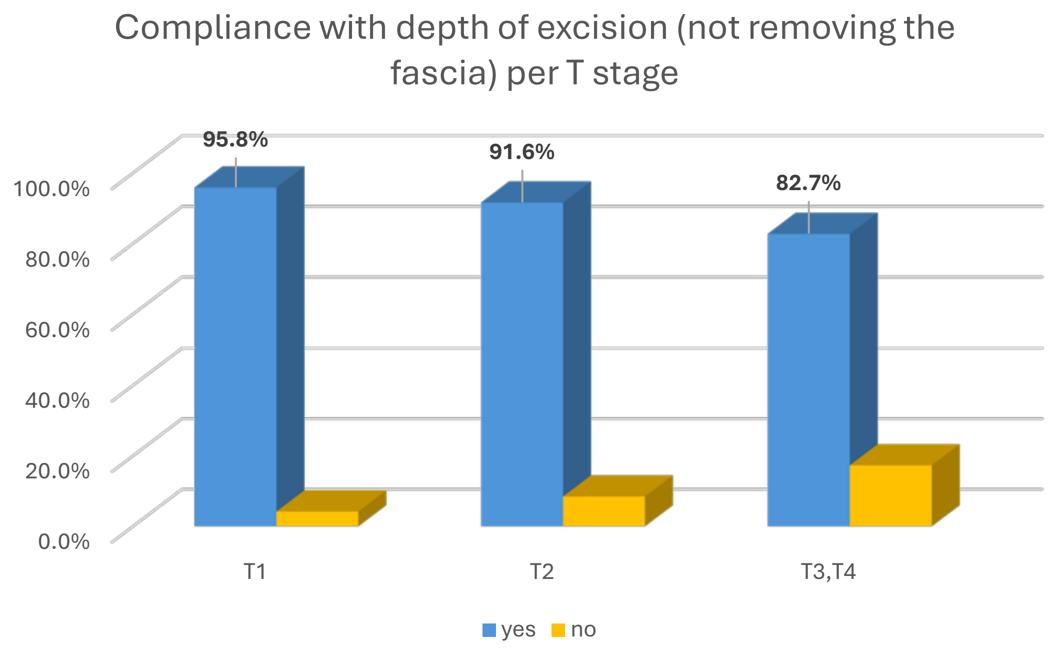
Compliance with depth of excision (not removing the fascia) per T stage
95.8%
91.6%
82.7%
100.0%
80.0%
60.0%
40.0%
20.0%
0.0%
T1
T2
T3,T4
yes
no

B

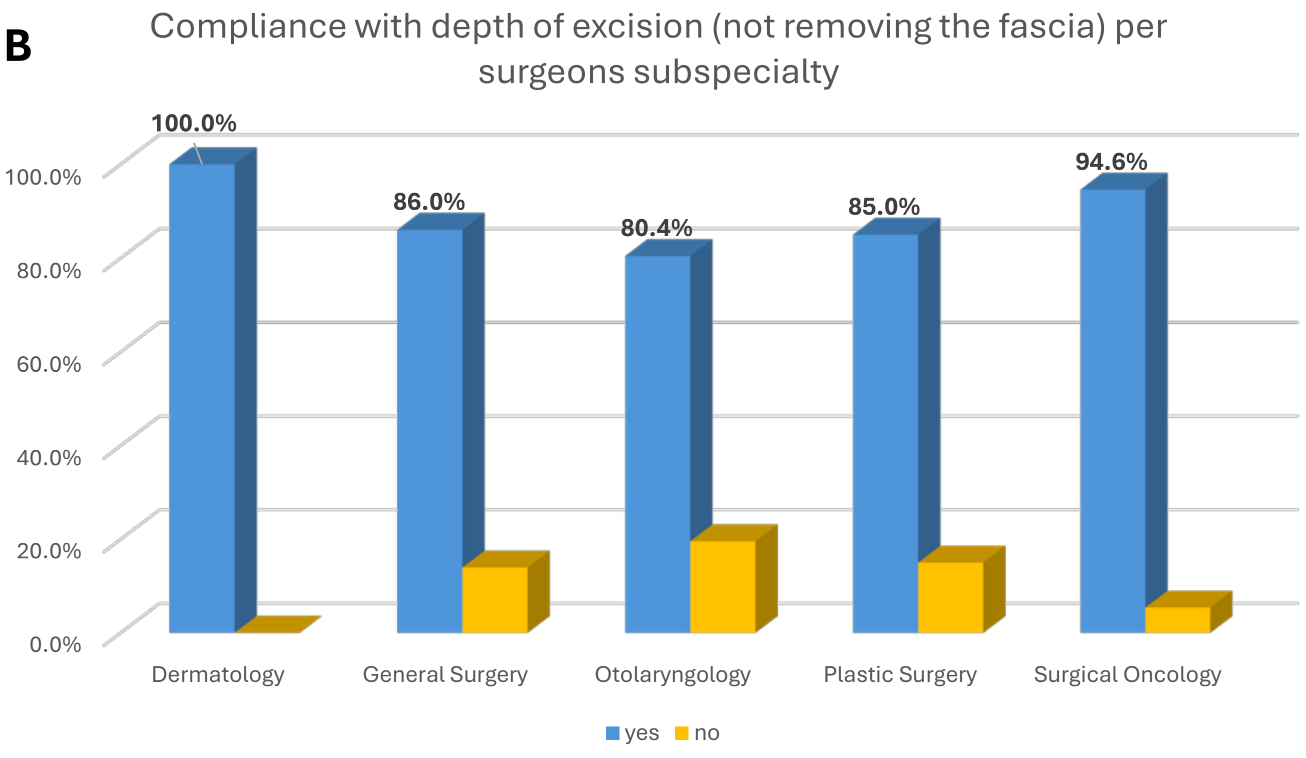
Compliance with depth of excision (not removing the fascia) per surgeons subspecialty
100.0%
86.0%
80.4%
85.0%
94.6%
100.0%
80.0%
60.0%
40.0%
20.0%
0.0%
Dermatology
General Surgery
Otolaryngology
Plastic Surgery
Surgical Oncology
yes
no
