# Supplementary material for: Variation in the Surgical Care of Early Stage Melanoma Based on Surgical Subspecialty: Evaluation of Large Healthcare System
Source: Ann Surg Open. 2026 Feb 9;7(1):e650. doi: 10.1097/AS9.0000000000000650 (PMC13016181; doi:10.1097/AS9.0000000000000650)
Supplement: Supplementary file 6 [file as9-7-e650-s006.pdf]

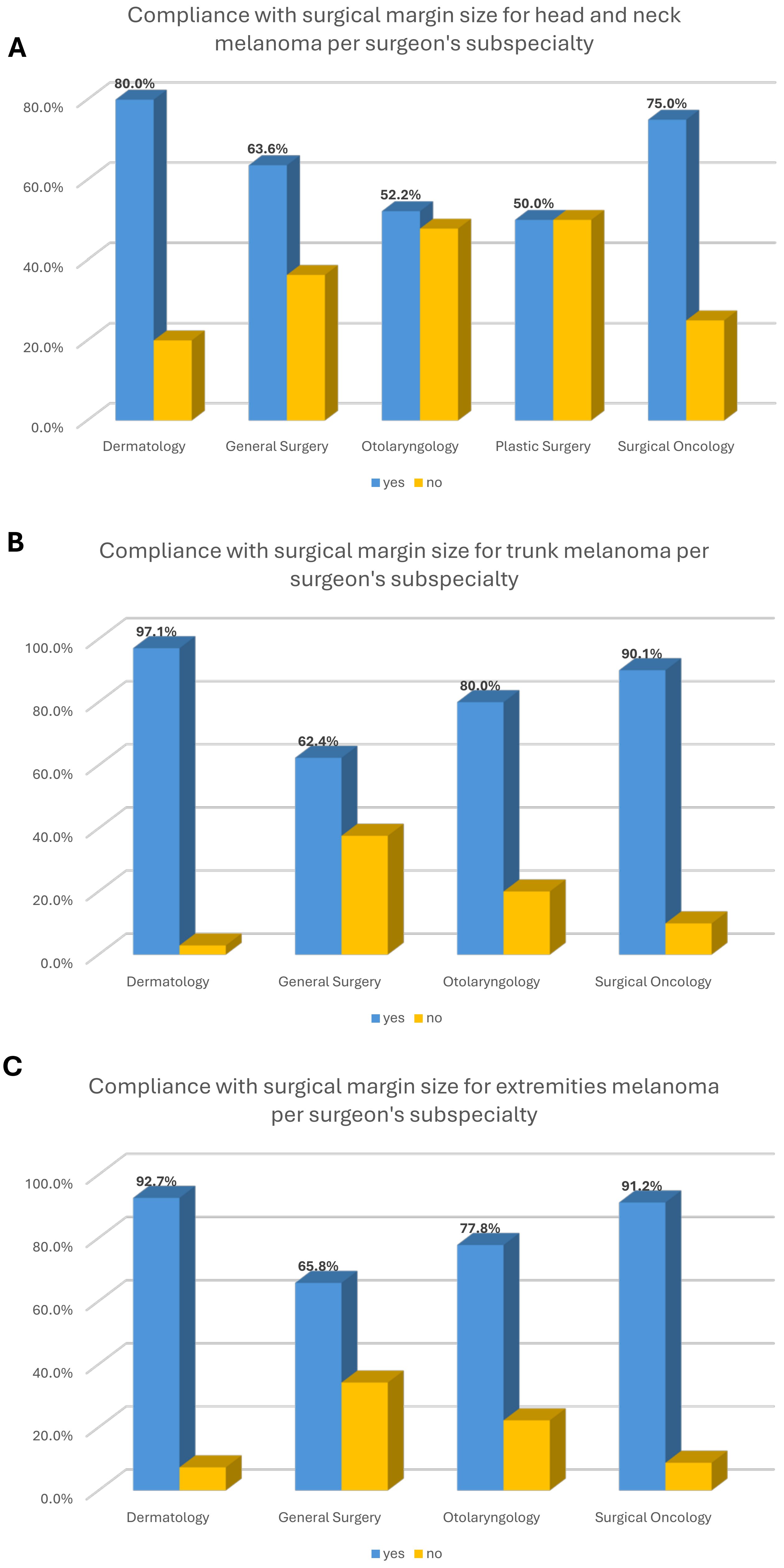
A
Compliance with surgical margin size for head and neck melanoma per surgeon's subspecialty
80.0%
60.0%
40.0%
20.0%
0.0%
80.0%
63.6%
52.2%
50.0%
75.0%
Dermatology
General Surgery
Otolaryngology
Plastic Surgery
Surgical Oncology
yes
no
B
Compliance with surgical margin size for trunk melanoma per surgeon's subspecialty
100.0%
80.0%
60.0%
40.0%
20.0%
0.0%
97.1%
62.4%
80.0%
90.1%
Dermatology
General Surgery
Otolaryngology
Surgical Oncology
yes
no
C
Compliance with surgical margin size for extremities melanoma per surgeon's subspecialty
100.0%
80.0%
60.0%
40.0%
20.0%
0.0%
92.7%
65.8%
77.8%
91.2%
Dermatology
General Surgery
Otolaryngology
Surgical Oncology
yes
no
